# Supplementary material for: Horizontal transmission maintains host specificity and codiversification of symbionts in a brood parasitic host
Source: Commun Biol. 2023 Nov 16;6:1171. doi: 10.1038/s42003-023-05535-1 (PMC10654585; doi:10.1038/s42003-023-05535-1)
Supplement: Supplementary file 2 — Supplementary Information [file 42003_2023_5535_MOESM2_ESM.pdf]

Horizontal transmission maintains host specificity and codiversification of symbionts in a brood parasitic host

Luiz Gustavo A. Pedroso; Pavel B. Klimov; Sergey V. Mironov; Barry M. OConnor; Henk R. Braig; Almir R. Pepato; Kevin P. Johnson; Qixin He; Fabio Akashi Hernandes

Supplementary Information

Supplementary Note 1: Field Samples

To investigate patterns of host switching of feather mites in the *M. bonariensis* system, we conducted a large-scale survey of *M. bonariensis* and its most common foster parent bird species (for example, *Turdus* spp., *Zonotrichia capensis*, *Sicalis* spp., *Pitangus sulphuratus*, *Tangara* (=*Thraupis*) spp.; see Supplementary Data 2 for a complete list) in different regions in Brazil. We sampled mites from live birds, dead birds, and bird skins from 6 museums[1,2]. Fieldwork was carried out in September–November of 2017 and 2018 in 10 Brazilian states. Bird captures have also been done throughout these years in partnership with the bird banding ornithological group of the São Paulo State University (UNESP) in Rio Claro. In the field, birds were captured using mist nets and had their feather mites collected by removing infested feathers (usually one primary and tertiary flight feather, plus random body feathers when no downy-mites were spotted) (permits MMA 57944-3 issued by the Ministry of Environment and Climate Change (MMA) of Brazil; and ethic approval CEUA 12/2017 issued by the Comissão de Ética de uso Animal of the São Paulo State University (UNESP)), preserving them in plastic vials filled with 96% ethanol and refrigerated (when possible), or stored in individual plastic bags for subsequent processing in the lab (yielding either live or dead mites; dead dried mites were still suitable for DNA extraction). In the lab, all field samples were visually inspected to identify different

morphospecies under a dissecting microscope; each morphospecies was then individually selected for DNA isolation and stored at -20 °C in 96% ethanol. Most mites collected in the field belonged to the genera commonly inhabiting New World passerines: *Amerodectes*, *Proctophyllodes*, *Metapterodectes*, *Tyrannidectes*, *Nycteridocaulus*, *Trouessartia*, *Analges*, *Mesalgoides*, and *Xolalgoides*[2,3].

Supplementary Note 2: Museum Samples

Feather mites usually remain on dead hosts and, therefore, could be sampled from dry bird skins in museums[2,4,5]. However, museum bird skins could be a source of cross-contamination between multiple bird species when they are processed by ornithologists in bulk (i.e. no individual bird separation), creating opportunities for mites to be transferred across adjacent bird specimens[2,6,7]. To account for possible museum cross-contamination, each mite species was ranked from 0 to 2 based on its estimated abundance (Supplementary Data 1: Column "Confidence Score"). Score 2 represents true mite-bird associations with high confidence (mites recovered from washing, from live birds in the field, or from museum skins with at least 3 males and 3 females of a given mite species found on a single host). Score 1 represents potentially true associations which failed the 3 male/3 female criterion. Score 1 is a realistic measure of mite associations because birds captured in the wild often have small mite infrapopulation sizes (e.g. only 2 specimens per bird can often be observed). Finally, score 0 represents likely contaminations (e.g., atypical, non-passerine mite species) or undersampled hosts. Their realized abundances may be affected by the skin preparation processes in museums, but this generally is not expected to be a substantial factor as mites remain firmly attached to the feathers even after their death.

Mites were sampled from six museums: Museu da Fundação Zoobotânica de Porto Alegre (MCN, Porto Alegre, Rio Grande do Sul); Museu de Ciências e Tecnologia da PUC (MCT, Porto Alegre, Rio Grande do Sul); Museu de História Natural Capão da Imbuia (MHNCI, Curitiba, Paraná); Museu de Zoologia da Universidade de São Paulo (MZUSP, São Paulo, São Paulo), Museu Paraense Emílio Goeldi (MPEG, Belém, Pará), and University of Michigan Museum of Zoology (UMMZ, Ann Arbor, Michigan), including the frozen tissue mite collection, which provided mites from *M. ater* and *Icterus pustulatus* for sequencing (Supplementary Data 2). Museum bird skins were sampled for mites by the feather-ruffling technique – bird's flight and body feathers were gently ruffled using a knitting needle over a clean, white paper; this material (debris, feather follicles, mites, etc.) was transferred to microtubes and then screened for mites in the lab under a dissecting microscope. Mites retrieved from museum skins were cleared in 30% lactic acid at 50°C for 48h before mounting on microscopic slides in Hoyer's medium; mites collected by washing and from live birds were left for 24h in lactic acid before mounting[8]. Mite exoskeletons retrieved from the non-destructive DNA extraction (see below) were placed in 30% lactic acid and briefly heated to remove internal air bubbles before mounting on slides. Mites were identified using the following references[2,9–14]. Sequential numbers (e.g. sp.1, sp.2) were assigned to undescribed species, which are common in the New World[3,15,16]. Slide-mounted mites were deposited at the Department of Ecology and Zoology of the Universidade Federal de Santa Catarina (ECZ–UFSC).

Supplementary Note 3: DNA Amplification and Sequencing

DNA was isolated from individual mites using the QIAmp DNA Micro Kit (Qiagen) following the manufacturer's protocol with the following modifications: (i) each mite was pierced using a

sterile pin under a dissecting microscope in a drop of ATL buffer and then transferred to a sterile microtube containing 180 µL ATL buffer and 20 µL of proteinase K, (ii) to avoid the mite being stuck to the tube wall, this solution was not pulse-vortexed, but (iii) was directly transferred to a shaking incubator (56° C, 24 h, lowest shaking speed), (iv) tubes were not pulse-vortexed after adding AL buffer but gently inverted several times to mix the reagents, (v) the DNA bound to the extraction column's silica membrane was eluted in 25 µL of AE buffer and incubated for 5 min before the last centrifugation step. A similar approach was described in[17,18]. After DNA isolation, mites' exoskeletons were mounted on microscopic slides and deposited (as morphological vouchers) at the Department of Ecology and Zoology of the Universidade Federal de Santa Catarina (ECZ—UFSC). Specimens labeled with BMOC codes used in this study are deposited in the University of Michigan Museum of Zoology (UMMZ). Both selected genes (CO1 and HSP70) were amplified using a nested PCR, which is appropriate for low concentration DNA templates. Our PCR mix (20 µL total volume) had 2.0 µL of 10x PCR buffer, 1.4 µL of 50 mM $MgSO_4$, 1.4 µL of dNTPs (10mM), 0.8 µL of each 10 µM primer, 0.08 µL of Platinum® *Taq* Polymerase (Invitrogen), and from 0.44 to 3.5 µL of DNA template, depending on DNA quality and concentration as assessed by a semiquantitative PCR using universally conserved eukaryotic primers for the 28S domain D9-10 rDNA: 28SV (GTAGCCAAATGCCTCGTCA) and 28SX (CACAATGATAGGAAGAGCC)[17]. Amplification of CO1 (F: TGTAAAACGACGGCCAGT; R: CAGGAAACAGCTATGACC) and HSP70 (F: TGYGTDGCHRTNATGGARG; R: GCCATYTTYTTRTANGCCATYTC) genes was done following previous published protocols for psoroptidian mites[17–22]. Amplicons from nested reactions were visualized on a 1.5% agarose gel and purified using Agencourt® AMPure® magnetic beads. Molecular work was done at the Molecular Lab of the Research Museums Center of the University of Michigan and sequenced at

the University of Michigan Sequencing Core (export permit SISGEN A056A93). Chromatograms were assembled and edited in GeneStudio 2.2.0.0 (Genestudio, Suwanee, GA, USA). Contigs were aligned in Mesquite v. 3.6 (Maddison & Maddison, 2018) using previous feather mite sequences as a reference[20]. A small number of sequences were truncated due to sequencing inconsistencies.

Supplementary Note 4: Divergence Time Estimation

Time calibration points follow those of a previous study[20], which estimated divergence times only for the family Proctophyllodidae. Therefore, we limited our estimations to this family. We performed two time divergence analyses, one using fossil-mite divergence times based on previous estimates[20], and for comparison, one using bird host diversification events as estimated in[23]. Both analyses used the same three nodes for calibration points as in[20] – the split of the Proctophyllodinae subfamily into the *Proctophyllodes* lineage and the *Nycteridocaulus* lineage, which corresponds to the oscine/suboscine avian split estimated at 45.1 Mya by fossil-mite data (sigma σ=3.0) and to 76.5 Mya by host divergence data (σ=3.0); the second calibration point refers to the diversification of the *thraupis* + *quadratus* *Proctophyllodes* groups, estimated as 25.3 Mya by fossil-mite data (σ=4.0), which corresponds to the 21 Mya (σ=2.85) dispersal to the New World of the bird superfamily Emberizoidea (also known as Nine-Primaried Oscines); and the third calibration point refers to the *Amerodectes* generic clade, estimated to be around 44.3 Mya (σ=3.5) by the fossil-mite data, which also corresponds to the Emberizoidea arrival in the New World (21 Mya, σ=2.85). Because different divergence times for Passeriformes have also been reported in the later works[24–28], we did another divergence time estimation analyses using data from[26], which estimated an earlier divergence time for the oscine/subsoscine split (around

44 Mya) and to the New World Emberizoidea clade (around 17.5 Mya). The mean and sigma for this calibration were set to 44 and 5.0 for oscine/suboscine split; and to 17.5 and 3.0 for the New World Emberizoidea, respectively.

Six independent BEAST runs were performed with a sampling frequency of 5000 and 50 million generations in the CIPRES science gateway[29]. Each run was inspected for convergence in Tracer v1.7.1 (Rambaut & Drummond, 2009); burn-in was 15-50%. LogCombiner v2.6.2 was used to combine MCMC stationary samples from the six independent runs using a single 50% burn-in threshold, and TreeAnnotator v2.6.1 was used to summarize the 12,504 postburnin trees into a maximum credibility tree with node heights calculated as median heights. The maximum credibility tree was visualized and edited in FigTree 1.4.4[30]. None of the runs using the[23] time calibration recovered either a good effective sample size (>200) or convergence and were therefore discarded, whereas all other runs presented ESSs above 200 and good convergence. Similarly, [20] reported difficulties in achieving convergence in 8 of 18 BEAST runs using a similar dataset.

Supplementary Note 5: Foster parents of *Molothrus-alien* mites

There were a total of 69 *Molothrus-alien* records (Table 1). Based on morphology and molecular evidence, 26 records of 8 mite species could be confirmed to originate from known shiny cowbird foster parents (see Supplementary Data 3): *Trouessartia sicaliae* from *Sicalis* spp.; *Analges* sp. 6, *Proctophyllodes* cf. *thraupis*, *Trouessartia* aff. *megaplax* and *Amerodectes bilineatus* from the sayaca tanager, *Tangara* (=*Thraupis*) *sayaca*; and *Trouessartia capensis*, *Analges ticotico*, and *Proctophyllodes carmenmirandae* from the rufous-collared sparrow, *Zonotrichia capensis*[33–39]. Unfortunately, the remaining records could not be assigned to any

known foster parent bird species due to insufficient knowledge of feather mite diversity in the Neotropic region[3,16].

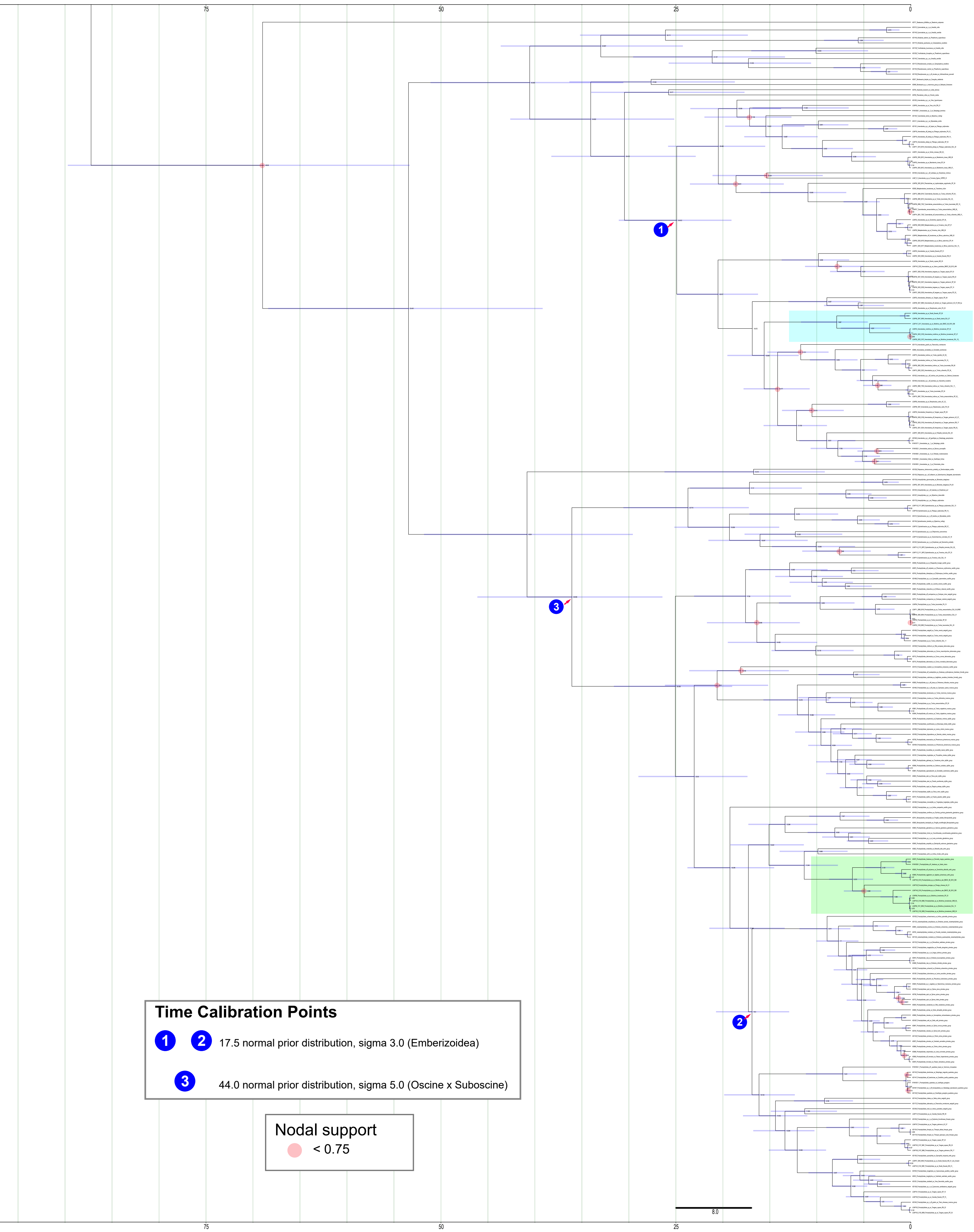

Supplementary Figure 1. Host calibrated divergence time estimates for the mite family Proctophyllodidae shown on a maximum credibility tree inferred in BEAST v2.6.1. For node calibration (points 1, 2 and 3), host phylobiogeographic information (Oliveros et al., 2019) was used. Median time estimates and their 95% highest posterior densities (bars) are given for each branch. Clades of interest are highlighted.

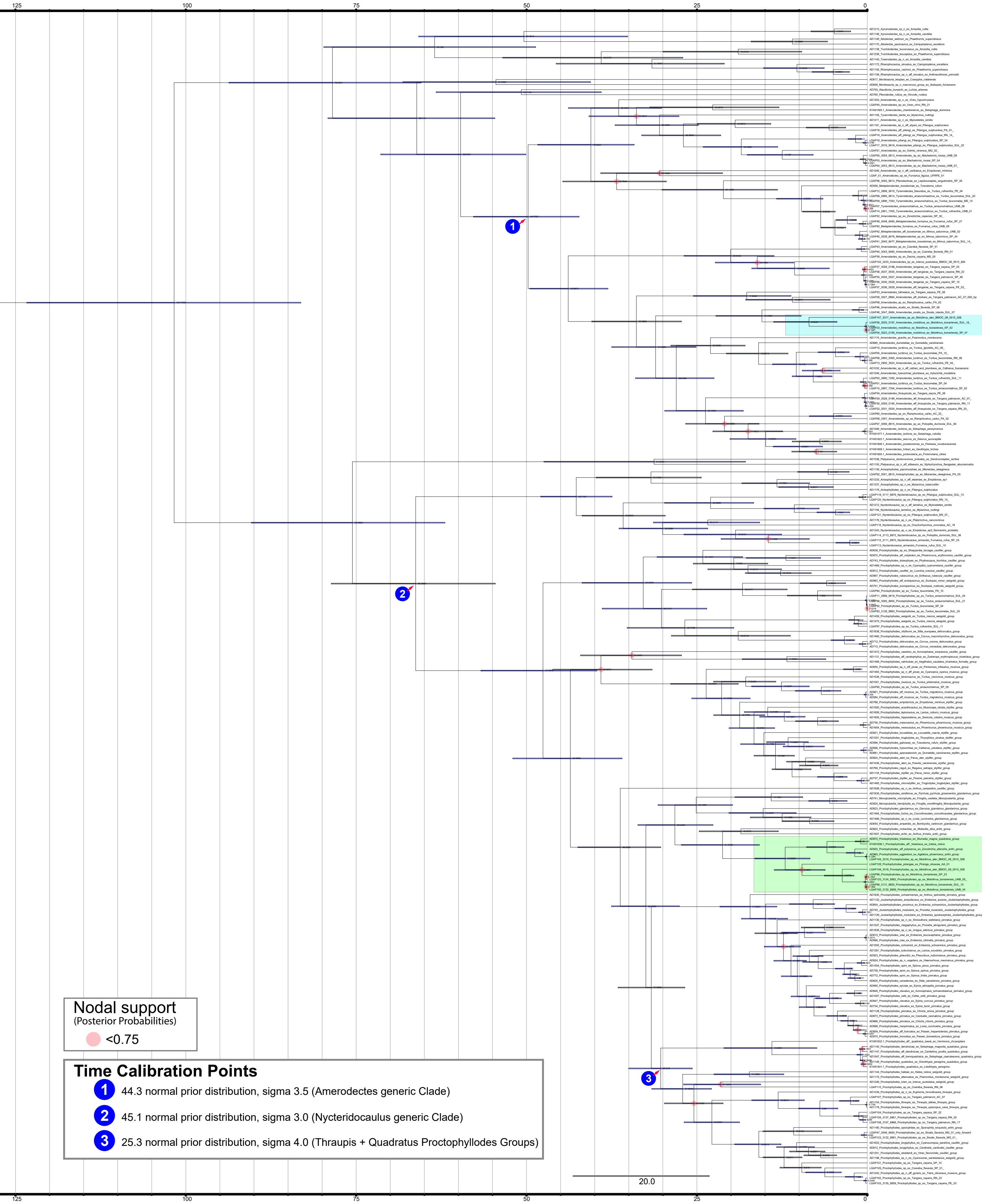

Supplementary Figure 2. Fossil mite calibrated divergence time estimates for the mite family Proctophyllodidae shown on a maximum credibility tree inferred in BEAST v2.6.1. For node calibration (points 1, 2 and 3), secondary calibration points inferred mite using mite fossil information previously (Klimov et al., 2017) (Points 1, 2 and 3). Median time estimates and their 95% highest posterior densities (bars) are given for each branch. Clades of interest are highlighted: light blue = *Amerodectes*; light green = *Proctophyllodes*.

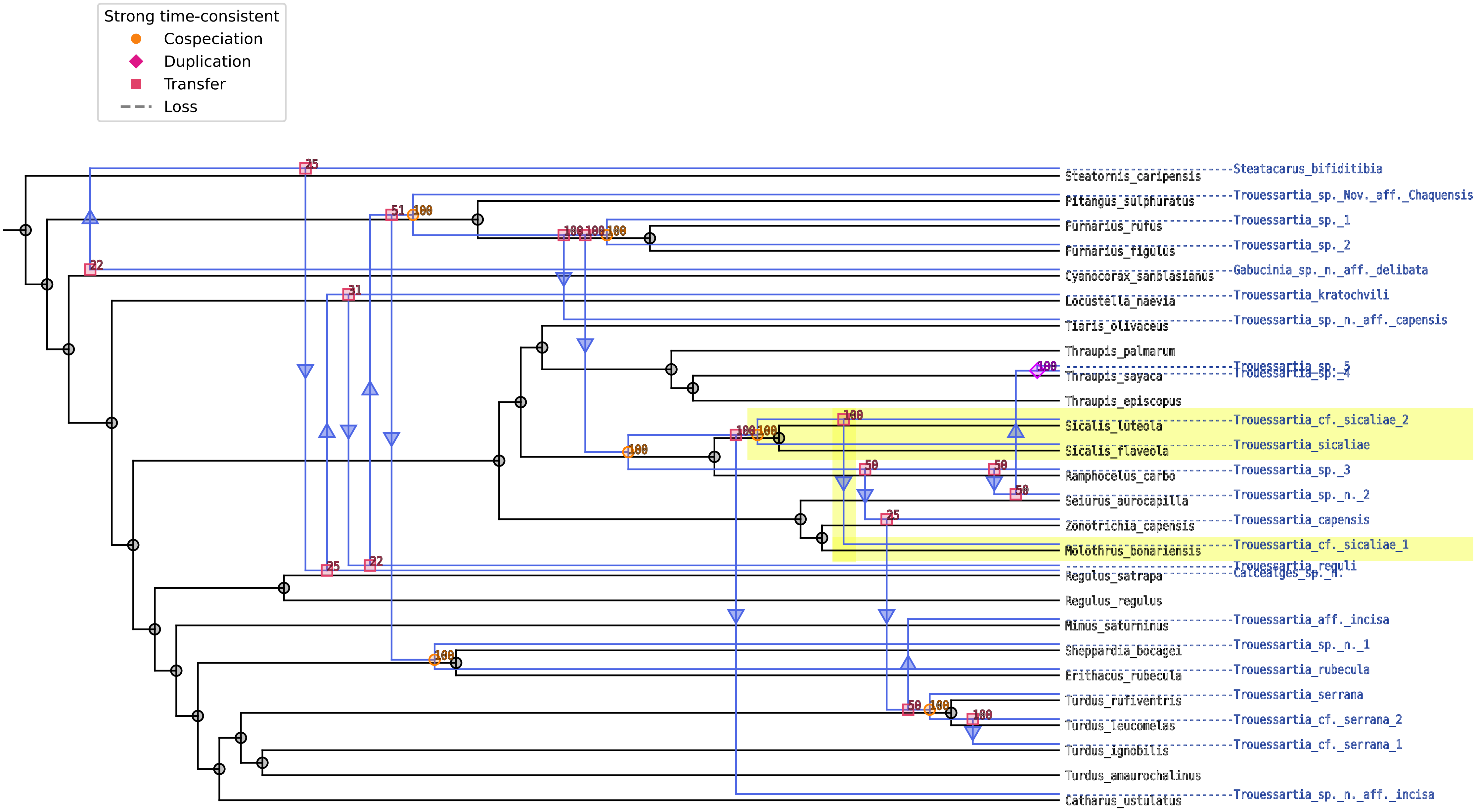

Supplementary Figure 3. Maximum parsimony cophylogenetic reconciliation for the mite family Trouessartiidae. Cophylogenetic events inferered for *Molothrus* and *Molothrus*-related mites are highlighted. See also Figure 3D. Event frequency values are given.

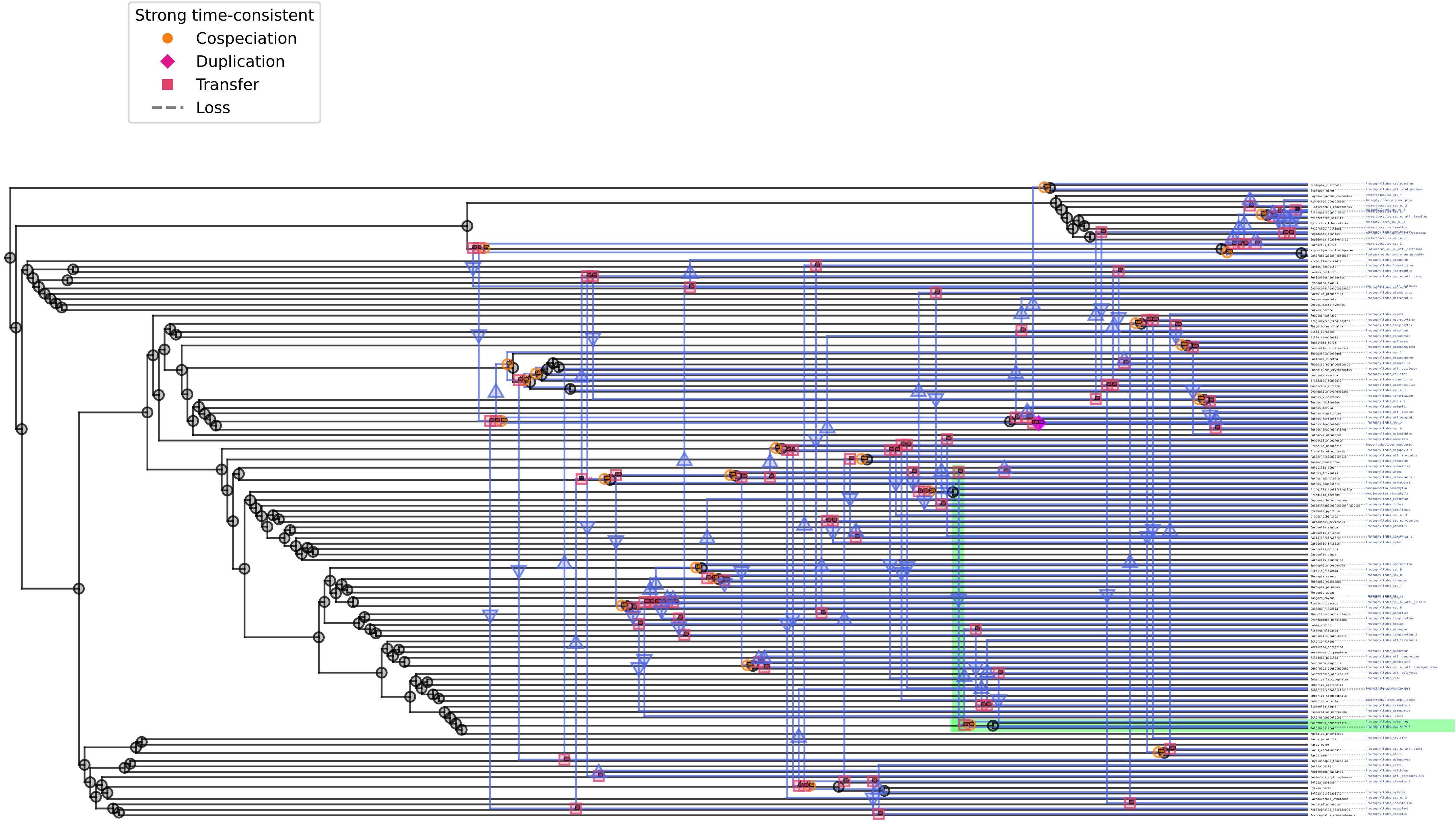

Supplementary Figure 4. Maximum parsimony cophylogenetic reconciliation for the mite subfamily Proctophyllodinae. Cophylogenetic events infered for *Molothrus* and *Molothrus*-related mites are highlighted. See also Figure 3B, C. Event frequency values are given.

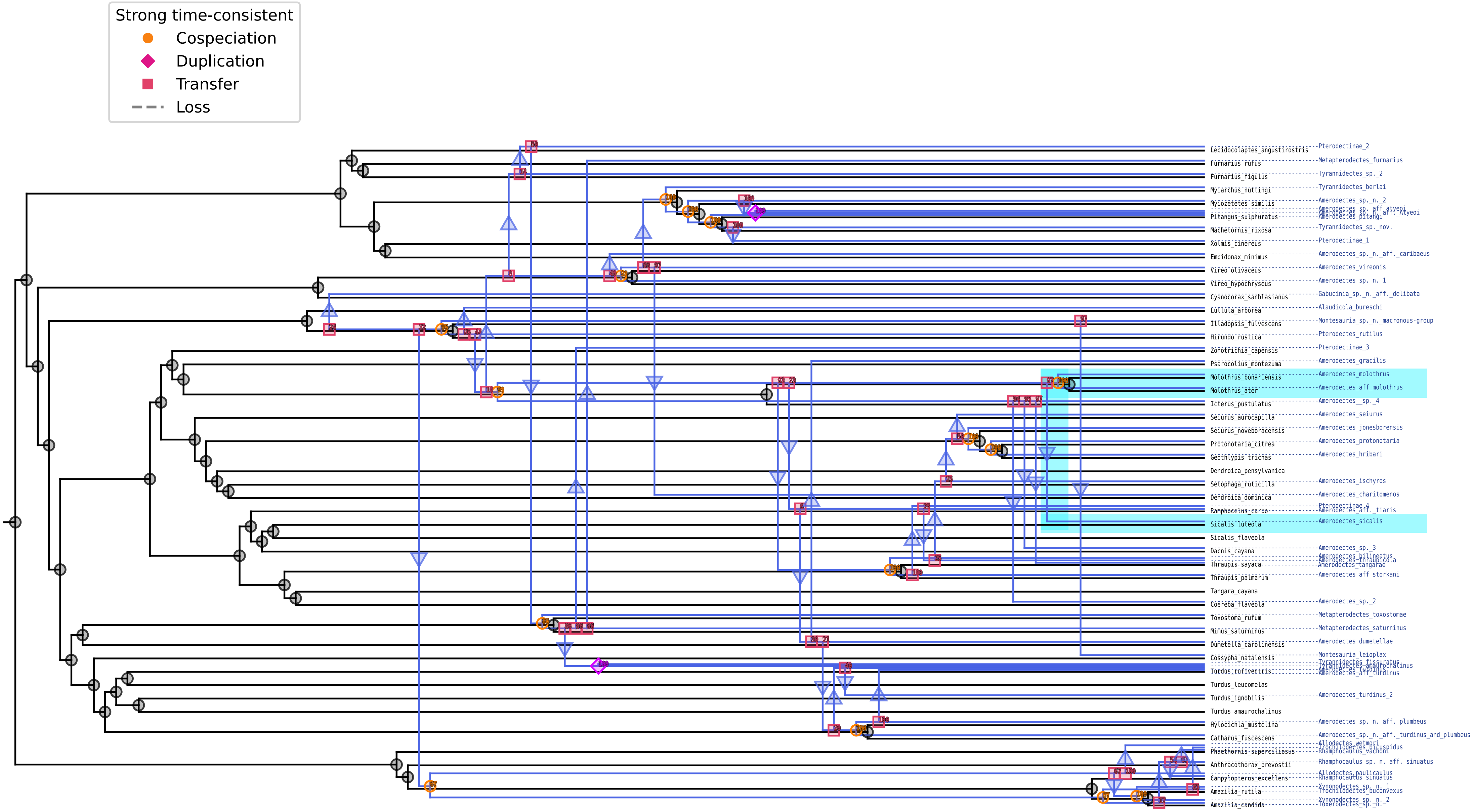

Supplementary File 5. Maximum parsimony cophylogenetic reconciliation for the mite subfamily Pterodectinae. Cophylogenetic events inferered for *Molothrus* and *Molothrus*-related mites are highlighted. See also Figure 3A. Event frequency values are given.

Supplementary References

1. Clayton, D. H. & Walther, B. A. Collecting and quantification of arthopod parasites of birds. *Host-parasites Evol. Gen. Princ. avian Model.* 420–440 (1997).
2. Gaud, J. & Atyeo, W. T. Feather mites of the world (Acarina, Astigmata): The supraspecific Taxa. *Ann. du Musée R. l'Afrique Cent. Sci. Zool.* **277** (Pt.1, 1-193 (text) & Pt. 2, 1-436 (illustrations) (1996).
3. Valim, M. P., Hernandes, F. A. & Proctor, H. C. Feather mites of Brazil (Acari: Astigmata: Analgoidea and Pterolichoidea). *Int. J. Acarol.* **37**, 293–324 (2011).
4. Hernandes, F. A. A review of the feather mite family Gabuciniidae Gaud & Atyeo (Acariformes: Astigmata: Pterolichoidea) of Brazil, with descriptions of eleven new species. *Zootaxa* **4747**, 1–53 (2020).
5. Mironov, S. V. & Proctor, H. C. The probable association of feather mites of the genus *Ingrassia* (Analgoidea: Xolalgidae) with the Blue Penguin *Eudyptula minor* (Aves: Sphenisciformes) in Australia. *J. Parasitol.* **94**, 1243–1248 (2008).
6. Choudhury, A., Moore, B. R. & Marques, F. L. P. Vernon Kellogg, host-switching, and cospeciation: Rescuing straggled ideas. *J. Parasitol.* **88**, 1045–1048 (2002).
7. Gaud, J. Acquisition d'hotes nouveaux par les Acariens plumicoles. *Bull. la Société Française Parasitol.* **10**, 79–91 (1992).
8. Krantz, G. W. & Walter, D. E. *A Manual of Acarology (3rd edition)* (eds. Krantz, G. W. & Walter, D. E.) 816 pp. (Lubbock: Texas Tech University Press, 2009).
9. Atyeo, W. T. & Braasch, N. L. The feather mite genus *Proctophyllodes* (Sarcoptiformes: Proctophyllodidae). *Bull. Univ. Nebrasca State Museum* **5**, 1–354 (1966).
10. Mironov, S. V., Literak, I. & Čapek, M. New feather mites of the subfamily Pterodectinae

(Acari: Astigmata: Proctophyllodidae) from passerines (Aves: Passeriformes) in Mato Grosso do Sul, Brazil. *Zootaxa* **38**, 1–38 (2008).

11. Mironov, S. V. New species of the feather mite genus *Proctophyllodes* Robin, 1877 (Acari: Analgoidea: Proctophyllodidae) from European passerines (Aves: Passeriformes), with an updated checklist of the genus. *Acarina* **20**, 130–158 (2012).

12. Pedroso, L. G. A. & Hernandes, F. A. Two new feather mites of the genus *Proctophyllodes* Robin (Acariformes: Proctophyllodinae) from passerines in Brazil. *Syst. Appl. Acarol.* **26**, 1081–1096 (2021).

13. Santana, F. J. A review of the genus Trouessartia (Analgoidea: Alloptidae). *J. Med. Entomol.* **1**, 1–128 (1976).

14. Valim, M. P. & Hernandes, F. A. A systematic review of feather mites of the *Pterodectes* generic complex (Acari: Proctophyllodidae: Pterodectinae) with redescriptions of species described by Vladimír Cerný. *Acarina* **18**, 3–35 (2010).

15. Barreto, M., Burbano, M. E., Proctor, H. C., Mironov, S. V. & Wauthy, G. Feather mites (Acariformes: Psoroptidia) from Colombia: Preliminary list with new records. *Zootaxa* **68**, 1–68 (2012).

16. Pedroso, L. G. A. & Hernandes, F. A. New records of feather mites (Acariformes: Astigmata) from non-passerine birds (Aves) in Brazil. *Check List* **12**, (2016).

17. Klimov, P. B. & OConnor, B. M. Origin and higher-level relationships of psoroptidian mites (Acari: Astigmata: Psoroptidia): Evidence from three nuclear genes. *Mol. Phylogenet. Evol.* **47**, 1135–1156 (2008).

18. Matthews, A. E. *et al.* Cophylogenetic assessment of New World warblers (Parulidae) and their symbiotic feather mites (Proctophyllodidae). *J. Avian Biol.* **49**, 1–17 (2018).

19. Bochkov, A. V., Klimov, P. B., Hestvik, G. & Saveljev, A. P. Integrated Bayesian species delimitation and morphological diagnostics of chorioptic mange mites (Acariformes: Psoroptidae: Chorioptes). *Parasitol. Res.* **113**, 2603–2627 (2014).
20. Klimov, P. B., Mironov, S. V. & OConnor, B. M. Detecting ancient codispersals and host shifts by double dating of host and parasite phylogenies: Application in proctophyllodid feather mites associated with passerine birds. *Evolution.* **71**, 2381–2397 (2017).
21. Klimov, P. B. & Oconnor, B. M. Is permanent parasitism reversible? - Critical evidence from early evolution of house dust mites. *Syst. Biol.* **62**, 411–423 (2013).
22. Knowles, L. L. & Klimov, P. B. Estimating phylogenetic relationships despite discordant gene trees across loci: The species tree of a diverse species group of feather mites (Acari: Proctophyllodidae). *Parasitology* **138**, 1750–1759 (2011).
23. Barker, F. K., Cibois, A., Schikler, P., Feinstein, J. & Cracraft, J. Phylogeny and diversification of the largest avian radiation. *Proc. Natl. Acad. Sci. U. S. A.* **101**, 11040–11045 (2004).
24. Barker, K., Burns, K. J., Klicka, J., Lanyon, S. M. & Lovette, I. J. New insights into New World biogeography: An integrated view from the phylogeny of blackbirds, cardinals, sparrows, tanagers, warblers, and allies. *Auk* **132**, 333–348 (2015).
25. Claramunt, S. & Cracraft, J. A new time tree reveals Earth history's imprint on the evolution of modern birds Supplemental Materials. *Sci. Adv.* 1–14 (2015) doi:10.1126/sciadv.1501005.
26. Oliveros, C. H. *et al.* Earth history and the passerine superradiation. *Proc. Natl. Acad. Sci. U. S. A.* **116**, 7916–7925 (2019).
27. Prum, R. O. *et al.* A comprehensive phylogeny of birds (Aves) using targeted next-

generation DNA sequencing. *Nature* **526**, 569–573 (2015).

28. Selvatti, A. P., Gonzaga, L. P. & Russo, C. A. de M. A Paleogene origin for crown passerines and the diversification of the Oscines in the New World. *Mol. Phylogenet. Evol.* **88**, 1–15 (2015).

29. Miller, M. A., Pfeiffer, W. & Schwartz, T. Creating the CIPRES Science Gateway for inference of large phylogenetic trees. *2010 Gatew. Comput. Environ. Work. GCE 2010* (2010) doi:10.1109/GCE.2010.5676129.

30. Rambaut, A. FigTree FigTree v1.4.4. *Institute of Evolutionary Biology, University of Edinburgh, Edinburgh. http://tree.bio.ed.ac.uk/software/figtree/* http://tree.bio.ed.ac.uk/software/figtree/ (2018).

31. Remsen, J. V., Powell, A. F. L. A., Schodde, R., Barker, F. K. & Lanyon, S. M. A revised classification of the Icteridae (Aves) based on DNA sequence data. *Zootaxa* **4093**, 285–292 (2016).

32. Gómez, R. O. & Lois-Milevicich, J. Phylogenetic signal in the skull of cowbirds (Icteridae) assessed by multivariate and cladistic approaches. *Zool. Anz.* **286**, 52–57 (2020).

33. Batisteli, A. F., da Silva Neto, E. N., Soares, T. P., Pizo, M. A. & Sarmento, H. Breeding biology of the Sayaca Tanager (*Thraupis sayaca*) in southeast Brazil. *J. Nat. Hist.* **53**, 2397–2412 (2019).

34. Cavalcanti, R. B. Shiny cowbird parasitism in central Brazil. *Condor* **90**, 40–43 (1988).

35. Fraga, R. M. The Rufous-Collared Sparrow as a Host of the Shiny Cowbird. *Wilson Bull.* **90**, 271–284 (1978).

36. King, J. Reproductive relationships of the Rufous-Collared Sparrow and the Shiny

Cowbird. *Auk* **90**, 19–34 (1973).

37. Lowther, P. E. Lists of victims and hosts of the parasitic cowbirds (*Molothrus*). *Field Museum of Natural History, Chicago, Illinois, USA URL* https://www.fieldmuseum.org/blog/brood-parasitism-host-lists (2019).

38. Mena, M. *et al.* Parasites of the Shiny Cowbird, *Molothrus bonariensis*, and the Austral Blackbird, *Curaeus curaeus*, (Passeriformes: Icteridae) in Chile. *Brazilian J. Vet. Parasitol.* **29**, 1–10 (2020).

39. Sick, H. *Ornitologia Brasileira (2nd ed.)* 862 pp. (Rio de Janeiro: Nova Fronteira, 1997).
