# Supplementary material for: Horizontal transmission maintains host specificity and codiversification of symbionts in a brood parasitic host
Source: Commun Biol. 2023 Nov 16;6:1171. doi: 10.1038/s42003-023-05535-1 (PMC10654585; doi:10.1038/s42003-023-05535-1)
Supplement: Supplementary file 3 — Description of Additional Supplementary Files [file 42003_2023_5535_MOESM3_ESM.pdf]

Description of Additional Supplementary Files

File name: Supplementary Data 1
Description: Molothrus bonariensis feather mite collection.

File name: Supplementary Data 2
Description: GenBank accession numbers, host and collection information

File name: Supplementary Data 3
Description: Putative Molothrus bonariensis foster parent hosts based on the identity of their feather mites (Molothrus-alien only).
