# Supplementary material for: Horizontal transmission maintains host specificity and codiversification of symbionts in a brood parasitic host
Source: Commun Biol. 2023 Nov 16;6:1171. doi: 10.1038/s42003-023-05535-1 (PMC10654585; doi:10.1038/s42003-023-05535-1)
Supplement: Supplementary file 7 — Reporting Summary [file 42003_2023_5535_MOESM7_ESM.pdf]

nature portfolio

Corresponding author(s): pklimov@purdue.edu

Last updated by author(s): Oct 16, 2023

# Reporting Summary

Nature Portfolio wishes to improve the reproducibility of the work that we publish. This form provides structure for consistency and transparency in reporting. For further information on Nature Portfolio policies, see our Editorial Policies and the Editorial Policy Checklist.

## Statistics

For all statistical analyses, confirm that the following items are present in the figure legend, table legend, main text, or Methods section.

| n/a | Confirmed | |
|---|---|---|
| ☐ | ☒ | The exact sample size (*n*) for each experimental group/condition, given as a discrete number and unit of measurement |
| ☐ | ☒ | A statement on whether measurements were taken from distinct samples or whether the same sample was measured repeatedly |
| ☐ | ☒ | The statistical test(s) used AND whether they are one- or two-sided<br>*Only common tests should be described solely by name; describe more complex techniques in the Methods section.* |
| ☒ | ☐ | A description of all covariates tested |
| ☒ | ☐ | A description of any assumptions or corrections, such as tests of normality and adjustment for multiple comparisons |
| ☒ | ☐ | A full description of the statistical parameters including central tendency (e.g. means) or other basic estimates (e.g. regression coefficient) AND variation (e.g. standard deviation) or associated estimates of uncertainty (e.g. confidence intervals) |
| ☒ | ☐ | For null hypothesis testing, the test statistic (e.g. *F*, *t*, *r*) with confidence intervals, effect sizes, degrees of freedom and *P* value noted<br>*Give P values as exact values whenever suitable.* |
| ☐ | ☒ | For Bayesian analysis, information on the choice of priors and Markov chain Monte Carlo settings |
| ☒ | ☐ | For hierarchical and complex designs, identification of the appropriate level for tests and full reporting of outcomes |
| ☒ | ☐ | Estimates of effect sizes (e.g. Cohen's *d*, Pearson's *r*), indicating how they were calculated |

*Our web collection on statistics for biologists contains articles on many of the points above.*

## Software and code

Policy information about availability of computer code

| | |
|---|---|
| Data collection | No software was used for data collection. |
| Data analysis | Graphic data analyses were conducted in the open source R environment version 3.6.3 (2020-02-29). The R packages used in this research to generated the graphics were: RColorBrewer v.1.1-2; ggpubr v.0.2.5; ggplot2 v.3.3.0. Phylogenetic analyses were performed using the softwares: GeneStudio 2.2.0.0; Mesquite v. 3.6; FigTree 1.4.4; BEAST v2.6.1; LogCombiner v2.6.2; TreeAnnotator v2.6.1; PartitionFinder v 2.1.1; IQ-Tree 1.6.10; and eMPRess. Additional analysis were also conducted in the websites BirdTree V2.ii (https://birdtree.org/), and ASAP (https://bioinfo.mnhn.fr/abi/public/asap/). |

For manuscripts utilizing custom algorithms or software that are central to the research but not yet described in published literature, software must be made available to editors and reviewers. We strongly encourage code deposition in a community repository (e.g. GitHub). See the Nature Portfolio guidelines for submitting code & software for further information.

## Data

Policy information about availability of data

All manuscripts must include a data availability statement. This statement should provide the following information, where applicable:

- Accession codes, unique identifiers, or web links for publicly available datasets
- A description of any restrictions on data availability
- For clinical datasets or third party data, please ensure that the statement adheres to our policy

DNA sequences were deposited into GenBank (accession MW814590–MW814707 and MW829221–MW829276). Supplementary Information provide the mite data and host (=Molothrus bonariensis) information (Supplementary Data 1); GenBank accession numbers, host and mite collection (Supplementary Data 2); mite collection data and putative foster parents for the host specificity categories: Molothrus-alien and QSM (Supplementary Data 3); Divergence time estimates using well-known host codivergence events (Supplementary Fig. 1) or mite outgroup fossil information (Supplementary Fig. 2); Maximum parsimony cophylogenetic reconciliations for the mite (sub)families: Trouessartiidae, Proctophyllodinae, Pterodectinae (Supplementary Fig. 3, 4, 5).

## Human research participants

Policy information about studies involving human research participants and Sex and Gender in Research.

| | |
|---|---|
| Reporting on sex and gender | N/A |
| Population characteristics | N/A |
| Recruitment | N/A |
| Ethics oversight | N/A |

Note that full information on the approval of the study protocol must also be provided in the manuscript.

# Field-specific reporting

Please select the one below that is the best fit for your research. If you are not sure, read the appropriate sections before making your selection.

☐ Life sciences ☐ Behavioural & social sciences ☒ Ecological, evolutionary & environmental sciences

For a reference copy of the document with all sections, see nature.com/documents/nr-reporting-summary-flat.pdf

# Ecological, evolutionary & environmental sciences study design

All studies must disclose on these points even when the disclosure is negative.

| | |
|---|---|
| Study description | We estimated and compared the transmission rates in an obligate host-symbiont system (feather mites on an obligate brood parasitic passerine, Molothrus bonariensis), by identifying different levels of host specificity. We also accounted for the effects of different transmission routes on the host specificity (microevolution scale) and cophylogenetic congruence (macroevolution scale) in the system by performing molecular phylogenies, time-calibrated phylogenies, and cophylogenetics analyses of feather mites on this host and on hosts commonly parasitized by M. bonariensis. |
| Research sample | The research sample consists of 144 specimens of Molothrus bonariensis (summing museum, captured in the field, and washed birds), plus 77 specimens from 29 species of passerines with molecular data in total (8 M. bonariensis samples overlapping, the others are 68 specimens of 27 species of putative M. bonariensis passerine foster parents, and 1 specimen of Molothrus ater). Data for M. bonariensis summed 365 mite records, and 1241 mites in total. New sequenced molecular data summed 118 samples in total. A list of samples and species is provided in Supplementary Data 1 and 2. |
| Sampling strategy | Natural History Museums and university-based scientific collections were targeted for the collection of feather mites on M. bonariensis scientific skins in Brazil and the USA (University of Michigan). Molecular-assessed feather mites were collected in partnership with ornithologists in different institutions in Brazil (a complete description is given in Supplementary Notes 1 and 2). Field samples were collected mostly in urban areas to maximize samplings of common passerine hosts used by M. bonariensis. |
| Data collection | Feather mites were sampled from museum bird skins by ruffling technique: each bird had all its feathers gently ruffled over a white paper, and the content was transferred to microtubes for later screening of mites in the lab. The collected mites were selected for microscopic slide mounting based on apparent morphologic differences under a dissecting scope. See Supplementary Note 2 for details. For field samples, birds were captured using mist nets and visually inspected for feather mites. Apparent mite-infested flight feathers and random body feathers were plucked from each bird and placed in individual zip-lock plastic bags. Feathers of apparent free-of-mite hosts were also plucked, which revealed in some cases to contain few mite specimens. The collected feathers were screened for feather mites later in the lab, and the mites were placed in microtubes containing 100% ethanol. See Supplementary Note 1 for details. Additional molecular data for feather mites were incorporated from Klimov et al. 2017 (doi:10.1111/evo.13309) |

| | |
|---|---|
| | for 6 genes, and from Matthews et al. 2018 (doi: 10.1111/jav.01580) for 1 gene. |
| Timing and spatial scale | Data collection occurred in 2017 and 2018 in Brazil, mostly during the bird's breeding season. Field samples were collected from September to October (2017) in the South Region in Brazil (States Rio Grande do Sul, and Paraná). From September to November (2018) in the North, Northeast, and Central-East in Brazil (States Acre, Pará, Rio Grande do Norte, Pernambuco, Brasília, Mato Grosso do Sul); in December 2018 in Minas Gerais State; and in different moments of these years in São Paulo State. The molecular data (DNA extraction, amplification, and sequencing) was processed in 2019-2020 at the Research Museum Center of the University of Michigan in Ann Arbor, USA, MI. |
| Data exclusions | Museum records were classified based on confidence scores: 0 (contamination), 1 (low confidence), and 2 (high confidence), in which 0 score samples were excluded from the analyses in order to account for possible cross-contamination of museum samples. See Supplementary Note 2. |
| Reproducibility | All data needed to reproduce our study are available in Data Availability. |
| Randomization | Field data collection was dictated by capture success of hosts. Museum host samples, when abundant, were chosen based on locality to cover for its geographic distribution. |
| Blinding | No blinding was necessary as this study involved the collection of raw data from natural history museum collections. |

Did the study involve field work? ☒ Yes ☐ No

## Field work, collection and transport

| | |
|---|---|
| Field conditions | Field work were performed mostly during the spring semester in Brazil (from September to November) in good weather conditions only. Except for collects done in Rio Claro, SP, where collect of feather mites were done throughout the years (2017-2018). |
| Location | Field samples were collected in 12 localities in Brazil: Unesp, Rio Claro, SP (22.39 S 47.54 W); Faz. 3 Irmãos, Rio Grande, RS (32.14 S 52.29 W); UFPR, Curitiba, PR (25.42 S 49.27 W); UEL, Londrina, PR (23.19 S 51.11 W); UFMS, Tres Lagoas, MS (20.47 S 51.39 W); UFPE, Recife, PE (8.02 S 34.57 W); UNB, Brasilia, DF (15.76 S 47.86 W); UFAC, Rio Branco, AC (9.57 S 67.44 W); MPEG, Belem, PA (14.42 S 48.44 W); UFRN, Natal, RN (5.50 S 35.12 W); UFMG, Belo Horizonte, MG (19.86 S 43.96 W); Porto Acre, AC (9.44 S 67.40 W). |
| Access & import/export | Access and collection of samples was conducted under permits MMA 57944-3 issued by the Ministry of Environment and Climate Change (MMA) of Brazil (25.04.2017), Ethic approval CEUA 12/2017 issued by the Comissão de Ética de uso Animal of the São Paulo State University (UNESP) (19.05.2017). Export of samples authorization number A056A93, issued by Sistema Nacional de Gestão do Patrimônio Genético (SISGEN) (16.04.2019). |
| Disturbance | After sampling, all birds were released back to the sites they were captured with little to no apparent disturbance. |

# Reporting for specific materials, systems and methods

We require information from authors about some types of materials, experimental systems and methods used in many studies. Here, indicate whether each material, system or method listed is relevant to your study. If you are not sure if a list item applies to your research, read the appropriate section before selecting a response.

### Materials & experimental systems

| n/a | Involved in the study | |
|---|---|---|
| ☒ | ☐ | Antibodies |
| ☒ | ☐ | Eukaryotic cell lines |
| ☒ | ☐ | Palaeontology and archaeology |
| ☐ | ☒ | Animals and other organisms |
| ☒ | ☐ | Clinical data |
| ☒ | ☐ | Dual use research of concern |

### Methods

| n/a | Involved in the study | |
|---|---|---|
| ☒ | ☐ | ChIP-seq |
| ☒ | ☐ | Flow cytometry |
| ☒ | ☐ | MRI-based neuroimaging |

## Animals and other research organisms

Policy information about studies involving animals; ARRIVE guidelines recommended for reporting animal research, and Sex and Gender in Research

| | |
|---|---|
| Laboratory animals | No laboratory animals were used in the present study. |
| Wild animals | For this study 77 specimens from 29 species of passerines from 10 families (Turdidae, Tyrannidae, Passerellidae, Icteridae, Thraupidae, Mimidae, Vireonidae, Furnariidae, Rhynchocyclidae, and Polioptilidae) were collected alive using mist nets and had their |

| | |
|---|---|
| | feathers plucked to access their feather mites. A complete list of bird and feather mite species used in the present study is provided in Supplementary Data 2. In field, birds were kept in individual bags until processing (collecting feathers for collecting mites and banding) and released afterward. |
| Reporting on sex | Sex was not considered in this study and the sex information was not uniformly collected as in most cases sex was not indicated in museum bird skins, nor the sex determined in the field. |
| Field-collected samples | A list of all bird species captured in field is given in Supplementary Data 2. The captured birds were kept in individual bags until processing (collecting feathers and banding) and released afterward. |
| Ethics oversight | Bird captures, handling, and feather collection were conducted following Brazil guidelines, under ethics approval number CEUA 12/2017 issued by the Comissão de Ética de Uso Animal of the São Paulo State University (UNESP) (19.05.2017). |

Note that full information on the approval of the study protocol must also be provided in the manuscript.
